# Korean Studies on Blood Stasis: An Overview

**DOI:** 10.1155/2015/316872

**Published:** 2015-03-02

**Authors:** Bongki Park, Sooseong You, Jeeyoun Jung, Ju Ah Lee, Kyung-Jin Yun, Myeong Soo Lee

**Affiliations:** Medical Research Division, Korea Institute of Oriental Medicine, Daejeon 305-811, Republic of Korea

## Abstract

Blood stasis is one of the important pathological concepts in Korean medicine. We analyzed the Korean studies concerning blood stasis. We searched for articles in eight electronic databases from their inception to September, 2014. We included reviews, clinical studies, and preclinical studies that had studied blood stasis and excluded articles in which blood stasis was not mentioned or in which the original authors had not explained blood stasis. Of 211 total included studies, 19 were reviews, 52 were clinical studies, and 140 were preclinical articles. “Stagnant blood within the body” was the most frequently mentioned phrase of the traditional concept of blood stasis. Traumatic injury was the most frequently studied disease/condition in the clinical studies. In the preclinical studies, coagulopathy was studied most frequently, followed by hyperviscosity, hyperlipidemia, inflammation, neoplasm, ischemic brain injury, and atherosclerosis. Hyeolbuchukeo-tang and Angelicae Gigantis Radix were the most frequent formula and single herb, respectively, used in the blood stasis researches. The results showed that blood stasis was mainly recognized as disorder of circulation and many studies showed the effectiveness of activating blood circulating herbs for diseases and pathologies such as traumatic injury or coagulopathy. Further studies are needed in the pathologic mechanisms and various diseases of blood stasis.

## 1. Introduction

Blood stasis is one of the important pathological concepts in traditional East Asian medicine (TEAM) since its concept was first documented in Huangdi's Inner Classic. Additionally, it is one of the currently active and vibrant research domains in TEAM [1, 2]. Generally, blood stasis is a significant pathological product due to stagnant blood [1, 2]. If blood stasis occurs within the body, known as “blood stasis syndrome (BSS),” characteristic symptoms such as pain in a fixed position, nyctalgia, dark-purple coloring of the tongue or face, infraorbital darkness, sublingual varicosis, blood spots under the skin or tongue, or an astringent pulse can manifest [3]. In clinical practice, many diseases include these signs and symptoms, such as ischemic heart disease, cerebral vascular accident, diabetes mellitus, chronic gastritis, chronic renal failure, chronic hepatitis, trauma, and dysmenorrhea. In the view of TEAM, these diseases could be related to BSS [4].

In recent decades, many preclinical and clinical studies for blood stasis have been conducted, and the correlation of blood stasis with diseases or physiopathology has been revealed. Many herbal formulas and activating blood circulating (ABC) herbs such as Hyeolbuchukeo-tang (*血府逐瘀湯*), Gyezibokryeong-whan (*桂枝茯苓丸*), Dangkwisoo-san (*當歸鬚散*),* Paeonia suffruticosa, Salvia miltiorrhiza, Paeonia albiflora, Angelica gigas, Hirudo nipponica, *and many other herbs were shown to be significantly effective for various diseases, including cardiovascular disease, traumatic conditions, and dysmenorrhea [5–8]. Through* in vivo* models and clinical trials, BSS's pathological features in biological rheology, thrombus, inflammation, and endothelium-derived vasoactive factors were uncovered [9–14]. Recently, system biological studies have revealed the mechanisms of blood stasis [15, 16].

Blood stasis is also a major pathogenic factor, and therefore, many studies have been conducted regarding blood stasis in Korean medicine. There was one review for researches of blood stasis in Korea [17]. However, it had some limitations including lack of search strategy and search term and limited searched databases. Therefore, the aim of this review was to analyze the Korean researches on blood stasis comprehensively.

## 2. Methods

### 2.1. Inclusion and Exclusion Criteria

This review was intended for all types of studies for blood stasis, regardless of language and publication date. We included reviews, all types of clinical studies, and preclinical studies that had included blood stasis, for example, a review of the concept of blood stasis or a cross-sectional study to reveal the relationship between blood stasis and a disease/condition or the* in vivo* research, to examine the effectiveness of ABC herbs. We excluded articles in which blood stasis was not mentioned or the original authors had not explained blood stasis as well as preclinical studies that were conducted as* in vitro* experiments or contained errors in the results. There were no limitations with regard to participants in the clinical studies or the TEAM's interventions (e.g., herbs, acupuncture, moxibustion, or pharmacoacupuncture).

### 2.2. Search Methods

We searched for articles in the following six Korean databases that were published prior to September 2014: Oriental Medicine Advanced Searching Integrated System (http://oasis.kiom.re.kr), Korean Traditional Knowledge Portal (http://www.koreantk.com/ktkp2014/), National Digital Science Library (http://www.ndsl.kr/index.do), Research Information Sharing Service (http://www.riss.kr/index.do), the Korean Studies Information Service System (http://kiss.kstudy.com/), and KoreaMed (http://www.koreamed.org). Additionally, we also searched in Pubmed and EMBASE for including Korean studies published overseas.

The search was conducted in Korean or English using the following terms: “blood stasis” or “static blood” or “blood stagnation” or “resolving blood” or “activating blood” or “activate blood” or “dissipate stasis” or “dissipating stasis” or “break stasis” or “expel stasis.”

### 2.3. Study Selection and Data Extraction

Two authors (B. Park and S. You) assessed the titles and abstracts of articles returned by electronic databases and determined their eligibility for inclusion. Hard copies of the relevant articles were retrieved. Four review authors (B. Park, S. You, J. Jeong, and J. A Lee) read the articles and extracted the data from the articles as the following:

- basic information of all articles (year of publication, title, author, and study design),
- the major concepts of blood stasis in all articles,
- disease/condition, study design, and a brief conclusion in the clinical studies,
- disease/pathology and the significant indicators in the preclinical studies,
- herbal formula and single herb in clinical and preclinical articles,
- herbal formula and single herb in the clinical and preclinical articles.

In terms of study design, we classified the articles into review, clinical study, and preclinical study. In addition, clinical studies were classified into randomized controlled trial, nonrandomized controlled trial, cohort study, case-control study, cross-sectional study, and case report [18].

Two authors (B. Park and S. You) assessed the titles and abstracts of the articles returned by the electronic databases and determined their eligibility for inclusion. Hard copies of the relevant articles were retrieved. Four review authors (B. Park, S. You, J. Jeong, and J. A Lee) read the articles and extracted the data from the articles according to the predefined criteria. Consensus was reached by discussion in the case of discrepancy. When disagreements were not resolved by discussion, they were arbitrated by another author (M. S. Lee).

### 2.4. Relationship between the Concept of Blood Stasis with Diseases/Conditions and Pathologies

After extracting the diseases/conditions and pathologies from the articles, the authors discussed the relationship between the traditional concept of blood stasis with pathologies and diseases/conditions. Then, the authors drew a diagram by connecting the extracted data. We used the “Cytoscape version 3.1.1” (http://www.cytoscape.org/) to draw the diagram.

## 3. Results

### 3.1. Study Selection and Description

**Figure 1:**
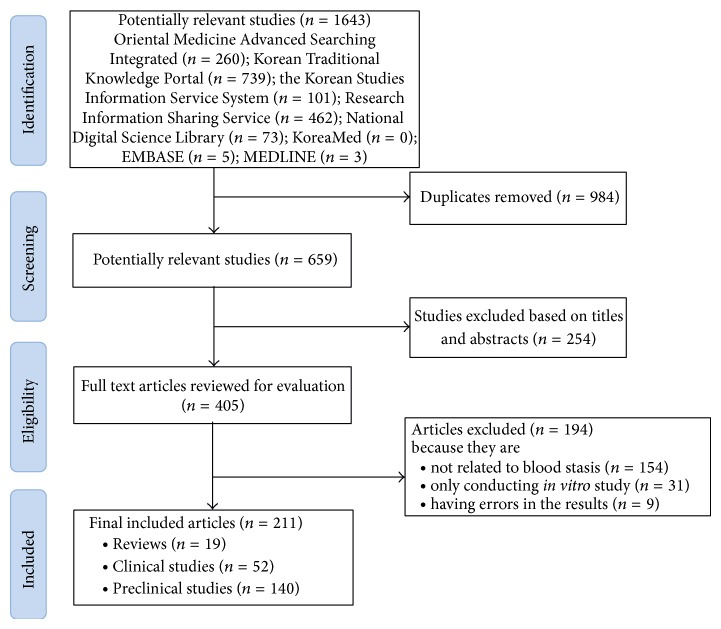
Flow diagram of study selection for qualitative analysis.

**Figure 2:**
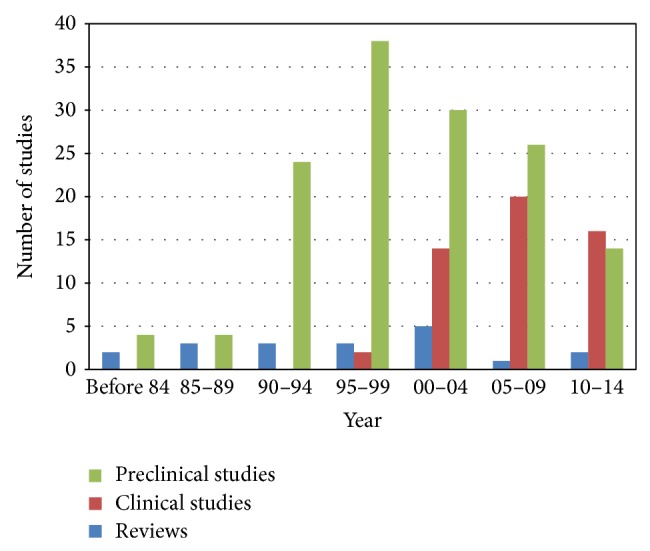
The number of articles according to the year of publication.

We screened a total of 659 articles by searching the 8 electronic databases, and 254 articles were excluded due to irrelevant titles and/or abstracts. The full texts of the remaining 405 articles were retrieved and evaluated. A total of 154 articles were excluded due to irrelevance to blood stasis, and a further 40 articles were excluded because 31 articles were only conducting* in vitro* study and 9 articles were an error in the results. In total, 211 articles were included in this review; 19 were reviews, 52 were clinical studies, and 140 were preclinical articles (Figure 1).  Figure 2 shows the number of articles according to the year of publication.

### 3.2. Concept of Blood Stasis in TEAM

After reading a total of 211 articles, we extracted the major concepts of blood stasis from each article. The major concept was extracted as a form of short paragraphs located in introduction or discussion sections of the articles. “Stagnant blood within the body” was the most frequently mentioned phrase regarding the blood stasis concept, followed by “disorder of blood circulation,” “pathological product,” “the blood lost its physiological function,” “pathogenic factor,” “extravasated blood,” “blood congested in viscera and tissue,” “foul blood,” “blood congested in a blood vessel,” “organ dysfunction,” and “stagnation of blood flow in local parts” (Table 1).

### 3.3. Disease/Condition and Study Design in the Clinical Studies

In the clinical studies, traumatic injury was the most frequently studied disease, followed by genitourinary tract disease, circulatory disease, and others (Table 1). Among the clinical studies, there were 24 case reports, 1 case-control study, 15 cross-sectional studies, 8 nonrandomized clinical trials, and 4 randomized clinical trials.

### 3.4. Pathology/Disease and Significant Indicators in the Preclinical Studies

In the preclinical studies, coagulopathy was studied most frequently relating to blood stasis, followed by hyperviscosity, inflammation, hyperlipidemia, oxidant stress, pain, liver injury, traumatic injury, neoplasm, ischemic brain injury, atherosclerosis, hypertension, diabetes mellitus, nephropathy, and hematoma (Table 1).

Among the 140 preclinical studies, we extracted indicators that were significantly improved or different between the treatment and control groups after herbal treatment (Table 2).

### 3.5. The Relationship between the Concept of Blood Stasis with Diseases/Conditions and Pathologies

**Figure 3:**
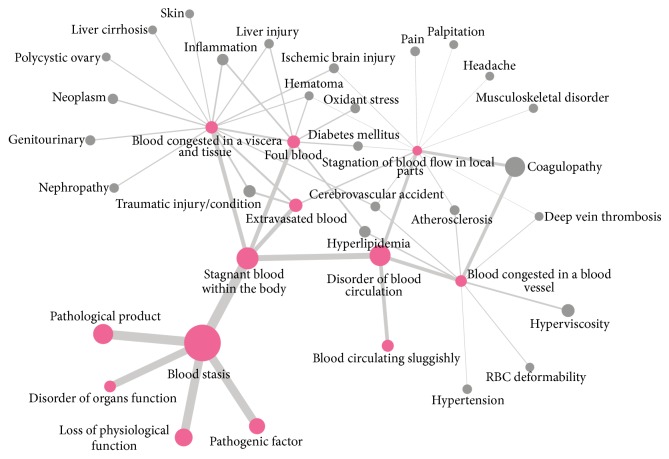
The relationship between the concept of blood stasis with pathology and diseases/conditions through the review. The sizes of the node, and line are emphasized by the frequency of quoted articles. The red nodes indicate the traditional concept of blood stasis.

Figure 3 is a diagram showing the relationship between the traditional concept of blood stasis with current pathologies and diseases/conditions.

### 3.6. Herbal Formula/Single Herb

The most frequently used herbal formula was Hyeolbuchukeo-tang (*血府逐瘀湯*, HCT), followed by Jungseongeohyeol-pharmacopuncture (中性*瘀血藥針*, JOP), Gyeokhachukeo-tang (膈下*逐瘀湯*, GCT), Gyezibokryeong-whan (*桂枝茯苓丸*, GBW), and Dangkwisoo-san (*當歸鬚散*, DSS) (Table 1). Angelicae Gigantis Radix was the most frequently used single herb in the blood stasis studies, followed by Persicae Semen, Carthami Flos, Paeoniae Radix Rubra, Cnidii Rhizoma, Glycyrrhizae Radix, Cyperi Rhizoma, Corydalis Tuber, Sappan Lignum, Achyranthis Radix, Moutan Cortex Radicis, and others (Table 1).

## 4. Discussion

This review overviewed the Korean studies on blood stasis. Regarding the traditional concept of blood stasis, “disorder of blood circulation” was the next common phrase after the “stagnant blood within the body.” As stagnant blood means almost the same thing as blood stasis, many researchers may mostly recognize blood stasis as disorder of circulation. It could explain the reason why coagulopathy was the most studied pathology relating to blood stasis, and many diseases/conditions and pathologies including hyperviscosity, ischemic brain injury, atherosclerosis, hypertension, and cerebrovascular accident were studied in connection with blood stasis. However, traumatic injury was the most studied disease/condition, followed by genitourinary disease in clinical studies. It may be related to the “extravasated blood” rather than “disorder of circulation.” We hypothesized that this ranking may be because many researchers thought that the previously mentioned diseases/conditions were more related to the blood stasis or were easily accessible research domains in Korea; both factors are likely contributors. Therefore, a larger variety of diseases related to the blood stasis should be studied in the clinical studies. Furthermore, most clinical studies focused on the effectiveness of ABC herbs in treating the disease or the correlation between blood stasis score and disease. It is necessary to analyze the biochemical difference in blood stasis patients compared with non-blood stasis patients in the same disease group or a healthy control group.

In the preclinical studies, most of the studies researched the ABC herbs' efficacy for coagulopathy, problems in blood vessels, and hyperviscosity. These pathologies are closely related to the notion of “disorder of blood circulation.” Considering the other notions of blood stasis and the clinical studies, the preclinical studies have been too heavily focused on the coagulopathy, the problems in blood vessels, and hyperviscosity. Furthermore, many studies only measured the indicators such as RBC, WBC, PLT, hematocrit, fibrinogen, fibrin degradation product, cholesterol, triglycerides, and viscosity and used similar preclinical designs. Although some studies showed interesting results for diabetic nephropathy and the mechanism of herbs for platelet aggregation [19–25], more in-depth studies are necessary to determine the molecular mechanisms.

The frequently used formulas in the blood stasis studies (HCT, GCT, GBW, and DDS) were also widely used for blood stasis clinically. HCT was mainly used in cardiovascular disease [26], GCT was used in liver disease [27], GBW was generally used in uterine disease [28], and DSS was used in traumatic injury [29]. JOP was manifested at the Korean Pharmacopuncture Institute, and it was used to treat diverse blood stasis diseases, particularly those involving musculoskeletal pain [30–32]. The concept of blood stasis was considered that it is stagnant blood within the body, which has lost its physiological function, and a pathological product as well as a pathogenic factor that causes organ dysfunction from the results. Stagnant blood within the body is indicated in disorder of blood circulation, extravasated blood, foul blood, and blood congested in viscera/tissue. In addition, disorder of blood circulation is including blood circulating sluggishly, blood congested in a blood vessel, and stagnation of blood flow in local parts. The diagram from our results simply shows the contents of Korean researches on blood stasis by connecting phrases of blood stasis concepts and each disease/condition and pathologies (Figure 3).

In summary, blood stasis was considered a significant pathogenic factor in Korean studies. The concept of blood stasis looked complicated, but we can organize the notions into four types: “disorder of blood circulation,” “extravasated blood,” “foul blood,” and “blood congested in viscera and tissue.” Many researchers mainly recognized blood stasis as disorder of circulation and most studies showed the effectiveness of ABC herbs for diseases and pathologies such as traumatic injury or coagulopathy. However, it is necessary to reveal the unique pathologic mechanisms of blood stasis and study the various blood stasis related diseases and the effectiveness of ABC herbs in more depth.

## Figures and Tables

**Table 1:** The concepts, diseases, and pathology regarding blood stasis in the reference articles.

	Total articles	Review articles	Clinical articles	Preclinical articles
Concept				

Stagnant blood within the body	97	16	7	18
Disorder of blood circulation	92	18	3	24
Pathological product	84	12	11	61
Loss of physiological function	67	8	8	51
Pathogenic factor	54	9	8	37
Extravasated blood	36	8	8	19
Foul blood	30	5	2	23
Blood congested in viscera and tissue	27	7	8	13
The blood circulating sluggishly	25	2	2	22
Blood congested in a blood vessel	21	5	3	13
Organ dysfunction	20	4		16
Stagnation of blood flow in local parts	6		1	5

Disease/condition/pathology				

Coagulopathy				91
Hyperviscosity				33
Inflammation				23
Hyperlipidemia				21
Oxidant stress				12
Pain				10
Liver injury				10
Traumatic injury			19	9
Neoplasm				9
Ischemic brain injury				9
Atherosclerosis				8
Hypertension				6
Diabetes mellitus				6
Nephropathy				6
Hematoma				2
Genitourinary			11	
Cerebrovascular accident			8	
Musculoskeletal disorder			4	
Etc.			8	

Herbal formula				

Hyeolbuchukeo-tang (*血府逐瘀湯*)	12		2	10
Jungseongeohyeol-pharmacopuncture (中性*瘀血藥針*)	9		5	4
Gyeokhachukeo-tang (膈下*逐瘀湯*)	6			6
Gyezibokryeong-whan (*桂枝茯苓丸*)	7		2	5
Dangkwisoo-san (*當歸鬚散*)	6		1	5

Herb				

Angelicae Gigantis Radix	107		19	88
Persicae Semen	99		22	77
Carthami Flos	84		19	65
Paeoniae Radix Rubra	82		20	62
Cnidii Rhizoma	79		14	65
Glycyrrhizae Radix	65		13	52
Cyperi Rhizoma	44		10	34
Corydalis Tuber	40		9	31
Sappan Lignum	35		12	23
Achyranthis Radix	34		9	25
Moutan Cortex Radicis	31		5	26
Salviae Miltiorrhizae Radix	30		8	22
Myrrha	29		9	20
Rehmanniae Radix	29		8	21
Linderae Radix	26		7	19

**Table 2:** The significant indicators investigated by ABC herbs in the preclinical studies.

Disease/pathology	Significant indicators (by ABC herbs)
Coagulopathy	RBC, WBC, PLT, hematocrit, fibrinogen, FDP, fibrinolytic activity, PLT aggregation, PT, aPTT, clotting time, prostaglandin synthase, PGE2, TBXA2, GPIIb/IIIa

Hyperviscosity	Whole blood viscosity, plasma viscosity

Hyperlipidemia	Total cholesterol, triglyceride, phospholipid, AST, ALT, HDL, LDL, VLDL, leptin, adiponectin, free fatty acid, lipoprotein

Inflammation	Edema, exudate, body weight, IL-1b, IL-6, IL-10, TNF-alpha, NO, PGE2, elastase activity, mast cell, WBC, MCP-1, COX-2

Neoplasm	Angiogenesis, pulmonary colonization of cancer cells, survival time, lymphocyte proliferation, NK cytotoxicity, apoptosis, tumor weight

Ischemic brain injury	Coma duration, mortality, ischemic area, edema area, neurologic grade

Atherosclerosis	Plaque size, histologic injury of aorta, lipid infiltration, membrane thickness

Traumatic injury	Neurological motor behavioral test, apoptosis, edema,

Oxidant stress	NO, ROS, superoxide, SOD

Pain	Response time, writhing syndrome

Hypertension	BP, dopamine, epinephrine, norepinephrine, NO, aldosterone, renin

Diabetes mellitus	Glucose

Nephropathy	24-hour urine protein, albumin, creatinine

Liver injury	ALP, LDL, LAP, AST, ALT, albumin

ABC: ALP: alkaline phosphatase; ALT: alanine transaminase; aPTT: activated partial thromboplastin time; AST: aspartate transaminase; BP: blood pressure; COX: cyclooxygenase; FDP: fibrin degradation product; GP: glycoprotein; HDL: high density lipoprotein; IL: interleukin; LAP: leucine amino-peptidase; LDL: low density lipoprotein; MCP-1: monocyte chemotactic protein-1; NK: natural killer; NO: nitric oxide; PGE2: prostaglandin E2; PLT: platelet; PT: prothrombin time; RBC: red blood cell; ROS: reactive oxygen species; SOD: superoxide dismutase; TBXA2: thromboxane A2; TNF: tumor necrosis factor; VLDL: very low density lipoprotein; WBC: white blood cell.
